# Polycystic ovary syndrome (PCOS) management using a nutrition recommender mobile application: identifying key requirements

**DOI:** 10.1186/s12905-025-03936-4

**Published:** 2025-08-14

**Authors:** Fahimeh Solat, Sharareh Rostam Niakan Kalhori, Goli Arji, Meysam Rahmani Katigari, Jebraeil Farzi, Mobina Zeinalabedini, Leila Shahmoradi, Leila Azadbakht

**Affiliations:** 1 Department of Health Information Management, Saveh University of Medical Sciences, Saveh, Markazi Iran; 2 Health Information Management and Medical Informatics Department, School of Allied Medical Sciences, Tehran University of Medical Sciences, Tehran, Iran; 3 Health Information Technology Department, School of Allied Medical Sciences, Zabol University of Medical Sciences, Zabol, Iran; 4 Department of Community Nutrition, School of Nutritional Sciences and Dietetics, Tehran University of Medical Sciences, Tehran, Iran; 5 Department of Clinical Nutrition, School of Nutritional Sciences and Dietetics, Shiraz University of Medical Sciences, Shiraz, Iran; 6 Endocrinology and Metabolism Research Centre, Tehran University of Medical Sciences, Tehran, Iran

## Abstract

**Objective:**

Polycystic Ovary Syndrome (PCOS) is a complex endocrine disorder that affects 5–18% of women worldwide. Adopting a healthier lifestyle, especially by making nutritious dietary choices, can significantly improve the management of symptoms and reduce complications associated with this condition. Despite the growing number of mobile health (mHealth) applications, most existing tools for PCOS lack scientific validation and dietary recommendations. This study aims to identify key requirements for developing a nutritional recommender mobile application for PCOS management.

**Materials and methods:**

This cross-sectional study was conducted in three stages: (1) Systematic review of articles and review of available mobile applications, (2) Data collection tool design, and (3) Validation of mobile application requirements based on the opinion of experts to provide nutritional recommendations for individuals with PCOS. (4) Design and evaluation of mobile application. Based on the results from the first stage, we created the questionnaire and assessed its validity based on the opinions of five experts in nutrition and dietetics, and health information management. Content modifications were made based on their opinions. The reliability was confirmed with a Cronbach’s alpha Coefficient of 74%. The questionnaire was distributed to seven PhD candidates and eight faculty members (from the Nutrition and Dietetics Department and the Health Information Management and Medical Informatics Department), and the results were analyzed and validated via SPSS 27 via CVR calculations.

**Results:**

In the first and second stages of the study, a total of 75 items were identified across four categories: educational needs (14 items), demographic information (nine items), data elements (32 items), and mobile application features (20 items). In the third stage, 56 items were considered high priority for inclusion in the mobile application based on their content validity ratio (49%< CVR).

**Conclusions:**

Considering the importance of self-care in patients with PCOS, the integration of a nutrition recommender mobile application can significantly enhance the lifestyle of individuals with PCOS, so it is essential to identify the requirements before conceptual design and application development for app usability and increasing user satisfaction.

## Introduction

Polycystic Ovary Syndrome (PCOS) is a complex hormonal disorder that affects 5–18% of women globally [1]. This syndrome is characterized by irregular ovulation, obesity, insulin resistance, and the formation of ovarian cysts [2]. In addition to increasing the risk of reproductive issues such as infertility, ovarian disorders, endometrial cancer, and early menopause, those affected by PCOS are also at heightened risk for mental health challenges, including depression, low self-confidence, and anxiety [3]. Furthermore, these patients may face a range of metabolic disorders, such as hypertension and cardiovascular diseases [4].

Changing patients’ lifestyles is considered the first step of treatment for these individuals. Regular physical activity, maintaining a healthy weight, avoiding smoking, and following a diet should be prioritized in lifestyle modifications [6]. A low Glycemic Index (GI) diet, a reduction in saturated fats, and increased fiber intake can effectively reduce inflammation and body fat while helping to stabilize blood sugar levels [7]. Therefore, adhering to a healthy eating pattern is crucial for regulating blood sugar and body fat, which helps manage this syndrome [5].

The use of mobile applications with recommendation features for addressing health-related issues, promoting lifestyle changes, and enhancing overall wellness has gained traction as a viable solution. Some apps focusing on diet management, physical activity tracking, and mental health support have shown positive effects on user engagement and health outcomes [21]. One of the technologies utilized in the healthcare field is the Clinical Decision Support System (CDSS), which assists various users in diagnosing and treating diseases [6]. A recommender system is a type of CDSS that simplifies the decision-making process by suggesting the best options based on the user’s preferences and individual circumstances [22]. The results of a previous study showed that CDSS has a positive impact on nutrition and quality of life for people with PCOS [5].

The design of mobile applications for customizable care is crucial, as these tools enable users to tailor care services based on their individual needs and preferences [8, 9]. This customization can encompass selecting schedules, types of services, and specific geographic areas [10, 11]. By utilizing user-friendly applications, individuals can easily and quickly respond to their needs, gaining a greater sense of control over the care they receive [12]. Additionally, analyzing the data collected through these applications can help service providers enhance the quality of services and improve the overall user experience. Therefore, well-designed applications not only improve user interaction with medical and caregiving services but can also lead to a better quality of life [13–16]. Thorough evaluation of mobile applications in the context of customizable care is essential, as this process helps identify both the strengths and weaknesses of the applications, fostering continuous improvement in user experience [17, 18]. Furthermore, evaluations can provide developers with valuable insights into the actual needs and preferences of users, enabling them to deliver better services and increase user satisfaction [19, 20].

Given the widespread use and capabilities of mobile phones in communities, developing a mobile application specifically for patients with polycystic ovary syndrome (PCOS) can be highly effective. However, many existing apps related to PCOS have faced criticism for lacking evidence-based content, expert involvement, and user validation [6]. So, we did four-step research. A systematic review we previously conducted (step one) found that although there are numerous programs available, few have been developed using rigorous frameworks or have been validated by stakeholders. Also, few of these provide dietary recommendations. To address these gaps, the current study focuses on steps two and three of a multi-step research plan. These steps involve data collection tool design and Validation of mobile application requirements on the basis of the opinions of experts specifically tailored to PCOS management. Unlike prior studies, this work combines expert knowledge with structured analysis to ensure that app development is more systematic and scientifically grounded. The validated requirements produced in this study will guide the future design and evaluation of a mobile application (step four) aimed at improving self-care and patient engagement for those with PCOS.

To address these issues, we conducted a four-step research process. In the first step, a systematic review revealed that while numerous programs are available, few have been developed using rigorous frameworks or validated by stakeholders. Additionally, very few of these apps provide dietary recommendations. The current study focuses on steps two and three of our multi-step research plans. These steps involve designing data collection tools and validating the mobile application requirements based on the opinions of experts specifically tailored to PCOS management. Unlike previous studies, this research combines expert knowledge with structured analysis to ensure that app development is systematic and scientifically grounded. The validated requirements produced in this study will guide the future design and evaluation of a mobile application aimed at improving self-care and patient engagement for individuals with PCOS.

## Related works

This study reviews various mHealth interventions aimed at promoting lifestyle changes, specifically for women with polycystic ovary syndrome (PCOS). The outcomes measured include Body Mass Index (BMI) as well as clinical, metabolic, and hormonal parameters. The findings indicate that following a healthy diet al.one can lead to a significant reduction in BMI. However, one major limitation of this study is the small number of studies available that demonstrate the effectiveness of lifestyle modifications in patients with PCOS, highlighting the need for further research to confirm these results [7]. The research also evaluates the impact of a smartphone app designed to assist with lifestyle modifications, focusing on dietary choices and exercise improvements for managing PCOS. According to the results, mobile applications that provide personalized support, accessibility, and affordability can help alleviate caregiving challenges for women with PCOS. Nevertheless, more research is required to explore the long-term effects of such interventions [8]. The primary objective of this study was to identify the essential features for applications aimed at training and facilitating self-care for patients with PCOS. To achieve this, we distributed a specially designed questionnaire among 45 patients and 17 doctors at the Fatemieh Educational and Medical Center in Hamedan. The limitations of the study included a restricted statistical population of patients from Hamedan and a lack of cooperation from some doctors and patients [9].

Recent surveys indicate that while self-care applications have been developed, there is a notable absence of tools specifically focused on modifying dietary habits and providing practical recommendations in Iran. Furthermore, most existing programs do not sufficiently emphasize nutrition.

## Materials and methods

**Fig. 1:**
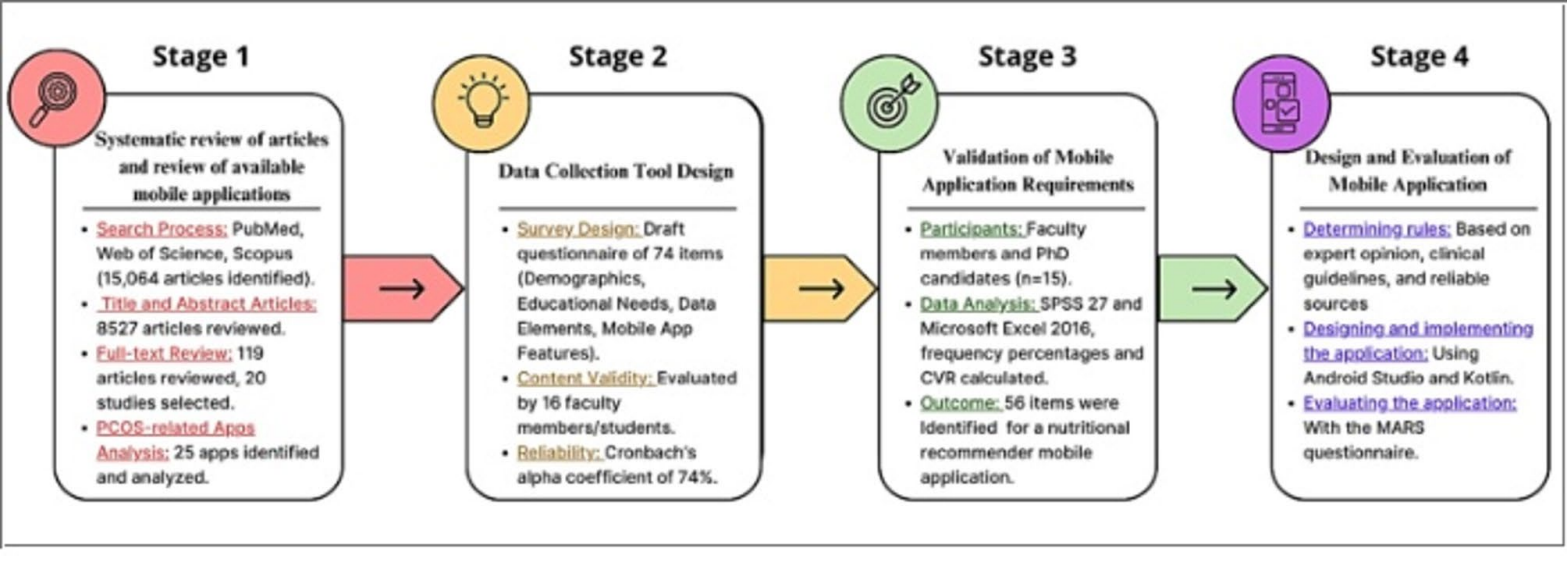
Study methodology at a glance

This cross-sectional study was conducted in three stages: (1) Systematic review of recommender systems [5] and available mobile applications, (2) Data collection tool design, and (3) Validation of mobile application requirements was performed on the basis of the opinions of experts to provide nutritional recommendations for individuals with PCOS (Fig. 1).

During the preparation of the study, the authors used “Grammarly” to fix grammar and writing problems. After using this tool, the authors reviewed and edited the content as needed and took full responsibility for the content of the publication.

### First stage: systematic review of articles and review of available mobile applications

A study titled “Nutritional Management Recommendation Systems in PCOS: A Systematic Review” was conducted at this stage. After defining the entry and exit criteria, the research team searched PubMed, Web of Science, and Scopus, identifying a total of 15,064 articles. Of these, 8,408 articles were excluded based on the inclusion and exclusion criteria. The remaining 119 articles underwent a full-text review, resulting in the selection of 20 studies. The research team extracted relevant data, including publication details and study findings [5]. Additionally, we examined 25 applications related to PCOS available on the Google Play Store, Galaxy Store, and Apple App Store. We searched for the terms “PCOS” and “polycystic ovary syndrome,” identifying and analyzing the 25 PCOS-related applications from these major app stores, while documenting their key features and relevance.

### Second stage: data collection tool design

Based on the results from the previous stage and feedback from experts, a questionnaire was created consisting of 75 items. We evaluated its validity by consulting five experts: two in nutrition and diet therapy and three in health information management at Tehran University of Medical Sciences. Content modifications were made based on their input. The reliability of all sections of the questionnaire was confirmed with a Cronbach’s alpha coefficient of 0.74.

### Third step: validation of mobile application requirements on the basis of the opinions of experts

At this stage, the study included six faculty members from health information management and medical informatics, two faculty members from nutrition and diet therapy, and seven PhD candidates in nutrition and diet therapy.

In needs assessment studies, particularly those utilizing purposive or convenience sampling, smaller sample sizes can effectively uncover key themes and user priorities [10]. Participants were selected for their direct experience with PCOS care and digital health, ensuring that the findings remain pertinent despite the limited number of participants.

Once the questionnaires were collected, the data were input into SPSS 27 and Microsoft Excel 2016. We calculated the frequency percentage and the Content Validity Ratio (CVR) for each item, removing any unnecessary items from the analysis. According to Lawshe’s criteria for CVR analysis, when there are 15 experts involved, the acceptable CVR for a data element is 0.49 [11]. Ultimately, we identified the requirements needed to design and develop a nutritional recommender mobile application.

### Fourth step: design and evaluation of mobile application

Based on the results of this study, a nutrition recommendation application has been developed for individuals with PCOS. The methodology used in this phase will be detailed in a future study. It is important to note that the application was designed using the programming language Kotlin and the Visual Studio Code editor.

## Results

### Systematic review of articles and review of available mobile applications

The characteristics of the studies included in the initial stage of our research have been documented in a systematic review article [5]. We examined 25 applications related to PCOS. The general and specific features of these applications are presented in Table 1. Overall, most of the applications focus on lifestyle modifications for individuals affected by PCOS, offering tools to monitor physical activity, nutrition, and sleep. With respect to food recommendations, the majority guided healthy eating through videos, text, and educational materials. However, only two applications offered a specific meal plan.

**Table 1:** General and specific characteristics of related applications

Name	Developer	Access link	Purpose			Year	Free or paid		Functional requirement	Thematic communication		
			Education	Diagnosis	Management		Free	In-app payment		Feeding	Self-care	Polycystic ovary syndrome
PCOS Tracker	Hello PCOS	https://pcostracker.app/	*		*	2019	*		Pedometer, Connecting to a smartwatch	*		*
Cysterhood	PCOS weight loss	https://pcosweightloss.org/enroll-cysterhood-membership/	*		*	2022		*	Communication with other patients, Gluten-free recipes, Sports exercises	*	*	*
PCOS	PCOS fertility nutrition	https://pcosfertilitynutrition.com/	*		*	2022	*		Educational materials about proper nutrition, stress control, and lifestyle in the form of text, podcast, and video	*	*	*
Uvi Health	Uvi health	https://uvihealth.in/	*		*	2021	*		Menstrual tracking, Consultation with Indian doctors, Interactive and live sports training, Meal plans	*	*	*
PCOS Diet	Edutainment ventures	https://www.edutainmentventures.com/	*		*	2022		*	Articles related to nutrition, cooking training channels, cooking video tutorials, Sleep and water drinking trackers, Pedometers, Calorie counters, BMI calculator	*		*
Social boat	Social boat	https://www.socialboat.live/			*	2022		*	Lose weight with a diet plan, weight tracking and Health recommendations	*		*
Cycle	Seashore	https://www.cicle.health/			*	2021		*	Menstrual period tracker, Monthly Calendar, Reminders			
Clue	Hello clue	https://helloclue.com/			*	2014		*	Menstrual period tracker, Pregnancy follow-up			
Ask PCOS	Ask PCOS	https://www.askpcos.org/		*	*	2021	*		The possibility of self-diagnosis of the disease, Tracking symptoms, personal dashboard			*
natural Cycles	FDA	https://www.naturalcycles.com/			*	2014	*		Body temperature tracking, ovulation and pregnancy tracker, menstrual cycle tracker			
Nabta	Nabta health	https://nabtahealth.com/			*	2020	*		Order a blood test at home, menstrual cycle management			
PCOS 1	Panchanite jas	https://drzio.com/	*		*	2021		*	Yoga training, daily diet tracker, Exercise reminder	*		*
PCOS And PCOD Help	OneLife2Care	https://www.onelife2care.com/			*	2021		*	Providing a daily meal plan	*		*
PCOS Revolution Lifestyle App	Trainerize	https://www.trainerize.com	*		*	2023	*		Sports training exercises, offer meals, Pressure reminder, Connect to wearable apps	*		*
Easy PCOS Diet Cookbook	Kevin Ton	https://play.google.com/store/apps/			*	2022		*	Display a list of the GI of foods	*		*
Ovie	The PCOS Nutritionist	https://thepcosnutritionist.com/ovie-app/	*		*	2023	*		Access to experts, interactive tests, dietary guidelines, providing sports exercises	*		*
PCOS Sisters Telehealth	Trainerize	https://pcossisters.trainerize.com/app/Logon.aspx		*	*	2022	*		Remote video visit, telephone consultation	*		*
PCOS	Manoj Upreti	https://freedomfrompcos.com/about-us.php				2021	*		-	*		*
Pollie	Pollie	https://www.pollie.co/	*		*	2023	*		Supportive care team, Access to a nutritionist, Track symptoms, training	*		*
Maya	Plackal	https://www.maya.live/			*	2011		*	Menstrual cycle tracking			
The PCOS Pt	Trainerize	https://thepcospt.trainerize.com/app/Logon.aspx			*	2022	*		Track meals and exercise	*		*
PCOS And Blessed	Trainerize	https://gracefulfitness.trainerize.com/app/Logon.aspx			*	2022	*		Track meals and exercise	*		*
Veera PCOS	Veeraheath	https://veerahealth.com/		*		2021	*		Online clinic	*		*
Clover	Wachanga	https://wachanga.com/			*	2018		*	Menstrual cycle calculator, reminder of the beginning and end of the cycle			
PCOS Diva	Kari Thorstenson	https://pcosdiva.com/app/			*	2023	*		Lifestyle modification programs, planning food supplements, automatic reminder	*		*

### Data collection tool design

The draft of the questionnaire included 75 items divided into four sections: demographic information (9 items), educational needs (14 items), data elements (32 items), and mobile application features (20 items). For the final version of the questionnaire, please refer to the supplementary file provided (Table S1).

### Validation of mobile application requirements on the basis of experts

**Fig. 2:**
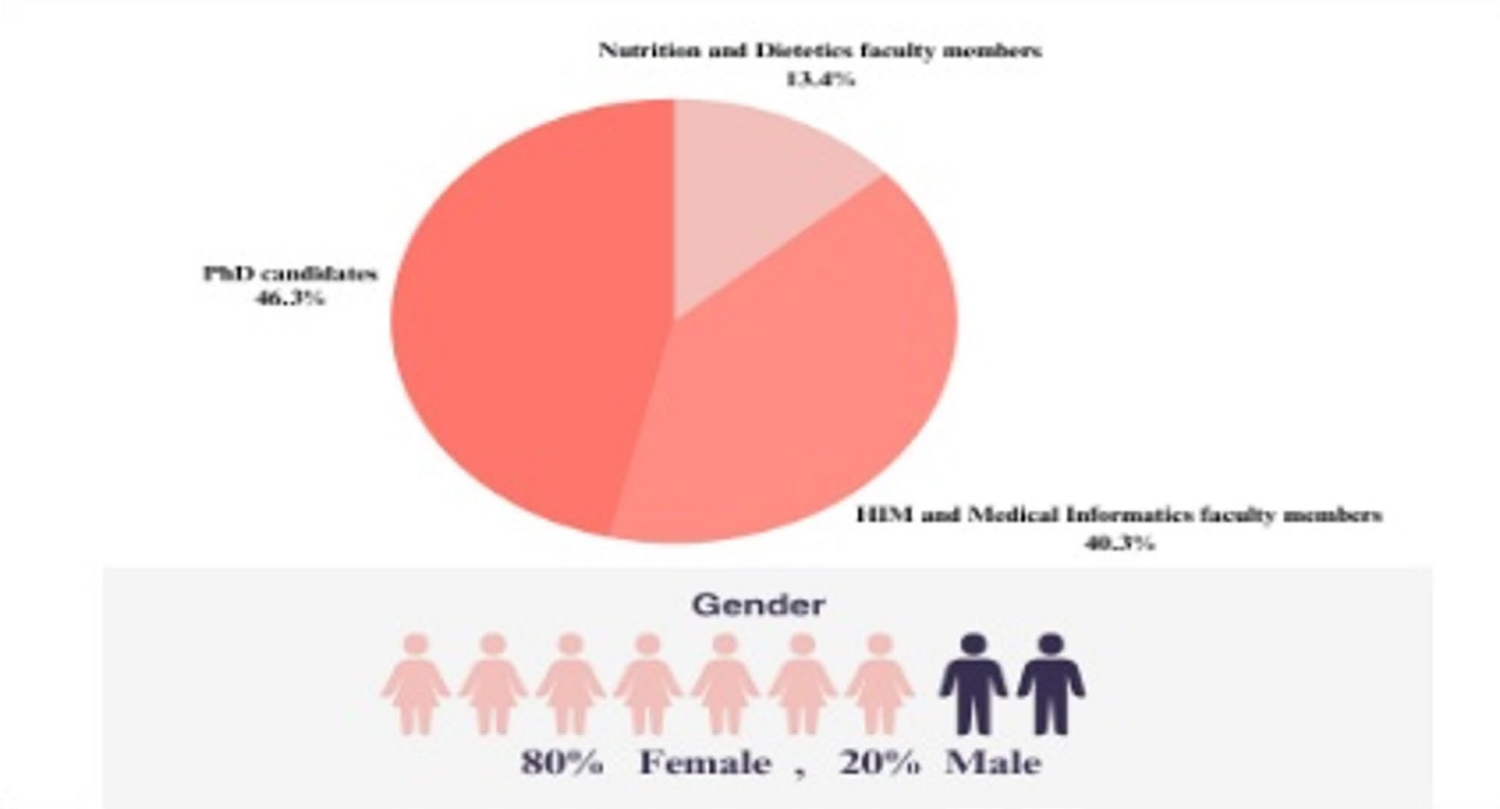
demographic characteristics of the respondents

The CVR was calculated for all the items. The items whose CVR was less than 0.49 were given a lower priority. The questionnaire was distributed to seven PhD candidates and eight faculty members: two (13%) from the Nutrition and Dietetics Department and six (40%) from the Health Information Management and Medical Informatics Department. Seven PhD candidates (46%) and all faculty members completed the questionnaire with satisfaction. Figure 2 presents the demographic characteristics of the respondents.

**Fig. 3:**
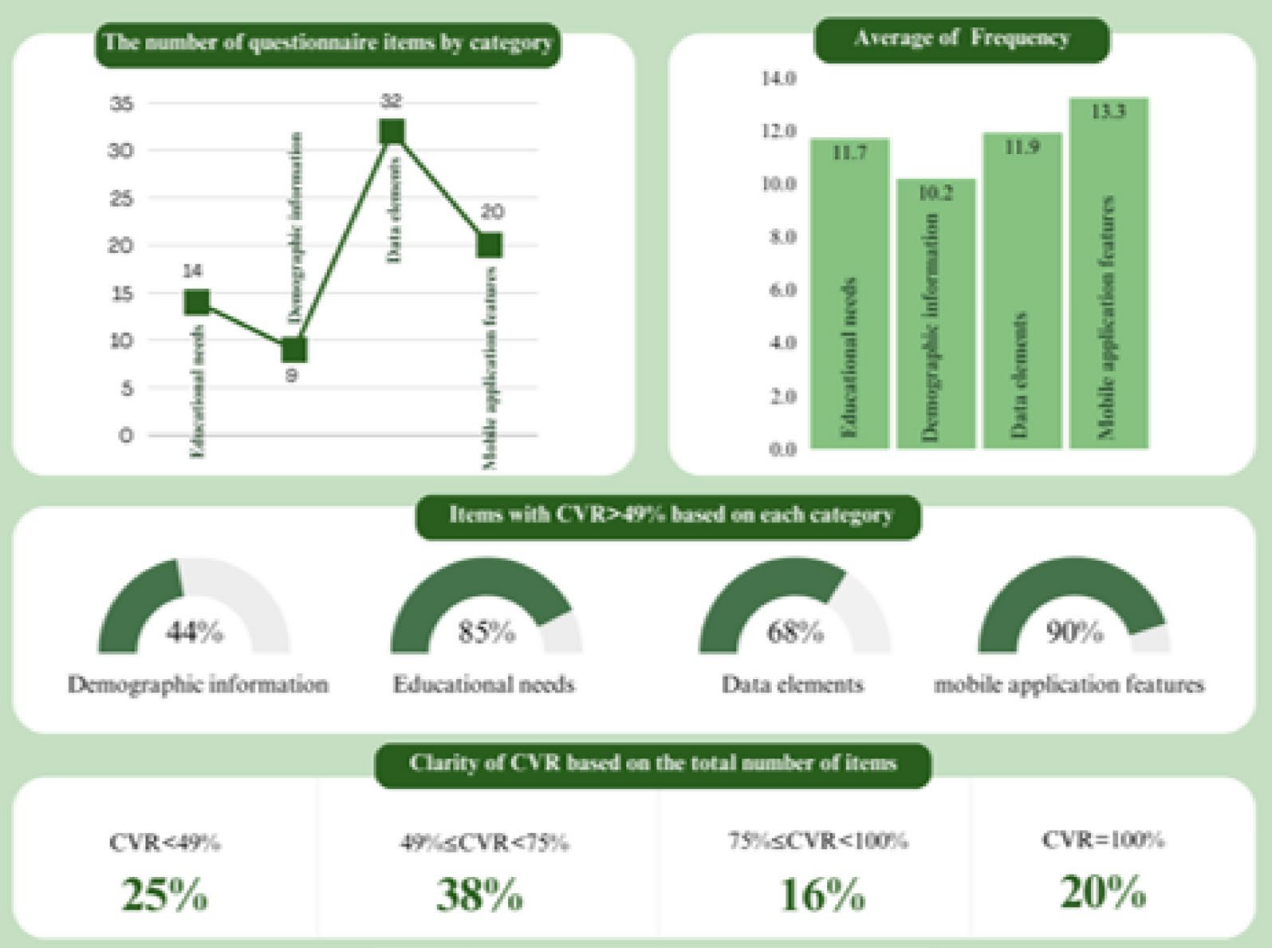
Analysis of the questionnaire data

In the second column of Table 2, the frequency and percentage of responses are listed According to respondents. Figure 3 shows the questionnaire results. 25% of the items had a CVR of less than 49%, which highlights the low necessity of these items to design and create mobile applications. Only 20% of the items were recognized as essential by all the experts (CVR = 100%). The other questionnaire items were in the range of 49–100%, which highlights the importance of these items.

In the category of **educational needs**, the average response frequency was 11.7. Among the 14 items assessed, 12 items (85%) were identified as highly necessary (with a Content Validity Ratio, or CVR, of 49% or higher). The items “news from Iran’s PCOS society” and “experiences of patients with PCOS” were classified as low necessity and therefore excluded.

The category of **demographic information** had the lowest average response frequency, at 10.2. Experts recognized five items (55%) in this category as low necessity, which were subsequently removed. However, the items “age,” “weight,” “height,” and “name and family name” had suitable CVR values (CVR ≥ 49%) and were selected for inclusion.

In the **data elements** category, the average frequency was 11.9. CVR calculations indicated that 22 items (69%) were deemed highly necessary for the design and development of the mobile application. Ten items (31%) were identified as low necessity and were removed.

The **mobile application features** category had the highest average frequency at 13.3, indicating strong consensus among the experts regarding these features. Out of 20 items in this category, 18 items (90%) had a CVR value of at least 49%, while items with CVR values below 49%, such as “ability to recommend medication” and “ability to recommend food supplements,” were considered low necessity and were excluded.

**Table 2:** Items extracted from the questionnaire

Category	Items of each category	CVR	Frequency (percentage)	low-necessity
Educational needs	PCOS and its types	73%	13 (87%)	
	Cause, symptoms of PCOS	73%	13 (87%)	
	Risk factors for PCOS	73%	13 (87%)	
	Common problems of PCOS patients	60%	12 (80%)	
	The importance of a healthy diet	100%	15 (100%)	
	Smoking	60%	12 (80%)	
	Fluid intake	70%	8 (53%)	
	The importance and purpose of self-care in illness	73%	13 (87%)	
	The importance of physical activity	100%	15 (100%)	
	The importance of stress control	60%	12 (80%)	
	News of Iran’s PCOS society	−20%	6 (40%)	*
	Ways to diagnose the disease	73%	13 (87%)	
	Types of disease treatment	73%	13 (87%)	
	Experiences of patients with PCOS	−20%	6 (40%)	*
Demographic information	Age	100%	15 (100%)	
	Weight	100%	15 (100%)	
	Height	100%	15 (100%)	
	Race	−33%	5 (33%)	*
	Place of residence	−7%	7 (47%)	*
	Job	20%	9 (60%)	*
	Level of education	20%	9 (60%)	*
	Name and family name	70%	8 (53%)	
	Contact number	20%	9 (60%)	*
Data elements	Blood group	33%	10 (67%)	*
	Existence of stress and anxiety	60%	12 (80%)	
	Heavy bleeding during menstruation	−7%	7 (47%)	*
	Duration of diagnosis	73%	13 (87%)	
	Patient symptoms (acne, facial hair, Hair loss, oily skin, the presence of dark spots skin)	87%	14 (93%)	
	Current medications and dosage	100%	15 (100%)	
	Drug sensitivity	100%	15 (100%)	
	Comorbidities such as diabetes and hypertension	87%	14 (93%)	
	Records of family diseases	73%	13 (87%)	
	Hospitalization records	100%	15 (100%)	
	Surgical interventions	100%	15 (100%)	
	Fluids consumed daily	87%	14 (93%)	
	Blood test history (LH, FSH, SHBG, PRL, PRGc)	70%	8 (53%)	
	Menstrual history	70%	8 (53%)	
	History of alcohol use	73%	13 (87%)	
	History of urinalysis	60%	12 (80%)	
	History of ovarian checkup	100%	15 (100%)	
	The size of the ovaries	33%	10 (67%)	*
	History of sports and physical activity	20%	9 (60%)	*
	History of smoking and tobacco use	100%	15 (100%)	
	History of heart rate and blood pressure	87%	14 (93%)	
	Body weight history	−7%	7 (47%)	*
	Blood glucose history	−7%	7 (47%)	*
	Record body temperature	20%	9 (60%)	*
	Daily urination	100%	15 (100%)	
	Food sensitivities	100%	15 (100%)	
	Fast food consumption	33%	10 (67%)	*
	Amount of fruit and vegetable consumption per day	47%	11 (73%)	*
	Food preferences	73%	13 (87%)	
	Favorite foods	87%	14 (93%)	
	Length of menstrual cycle	−7%	7 (47%)	*
	The amount of physical activity	73%	13 (87%)	
Mobile application features	Ability to recommend medication	47%	11 (73%)	*
	The ability to remember the drug used	73%	13 (87%)	
	Ability to recommend food supplements	33%	10 (67%)	*
	Ability to warn about drug side effects	73%	13 (87%)	
	Ability to recommend drug dosage	100%	15 (100%)	
	The ability to display the name of the forgotten drug	87%	14 (93%)	
	Ability to warn about drug interactions	87%	14 (93%)	
	Ability to recommend meals	87%	14 (93%)	
	The possibility of recommending the elimination of harmful foods	73%	13 (87%)	
	The possibility of recommending foods based on the glycemic index	87%	14 (93%)	
	Ability to recommend drinks	87%	14 (93%)	
	The possibility of recommending food alternatives	73%	13 (87%)	
	Ability to recommend healthy snacks	73%	13 (87%)	
	Ability to recommend meals based on weight	73%	13 (87%)	
	The possibility of recommending meals based on the symptoms of the disease	100%	15 (100%)	
	The possibility of communication with specialists and nutrition experts	87%	14 (93%)	
	Ability to store information on a central database	87%	14 (93%)	
	Ability to display cooking instructions	60%	12 (80%)	
	Ability to calculate BMI	73%	13 (87%)	
	Ability to count steps	73%	13 (87%)	

Figure 3 analyzes questionnaire items across four categories: Educational Needs, Demographic Information, Data Elements, and Mobile Application Features. Most items in the “Mobile Application Features” (90%) and “Educational Needs” (85%) categories were validated with a CVR above 49%. In contrast, only 44% of items in the “Demographic Information” category met this criterion. 25% of all items had a CVR below 49%, while 20% achieved full agreement with a CVR of 100%.

The Pearson correlation coefficient was used to measure the correlation between different categories of the questionnaire. The results shown in Table 3 indicate significant positive correlations among the four sections of the questionnaire. Section one exhibits statistically significant correlations with Category Two (*r* = 0.647), Category Three (*r* = 0.622), and Category Four (*r* = 0.620), all at the 0.05 significance level. This suggests that participants who scored higher in Category One also tended to score higher in the other categories.

Additionally, Category Two has a strong and significant correlation with Category Three (*r* = 0.685, *p* = 0.005), while its correlation with Category Four (*r* = 0.409, *p* = 0.130) is not statistically significant. This may indicate a conceptual or structural difference between Categories two and four that warrants further investigation or revision. Lastly, Category Three and Category Four are positively correlated with a significance level (*r* = 0.537, *p* = 0.039).

Overall, the results demonstrate acceptable internal consistency among most categories of the questionnaire; however, the non-significant correlation between Category Two and Category Four highlights a potential area for improvement.

**Table 3:** Correlations among the four categories of the questionnaire

		Average of Cat 1	Average of Cat 2	Average of Cat 3	Average of Cat 4
Average of Cat 1	Pearson Correlation	1	0.647*	0.622*	0.620*
	Sig. (2-tailed)	–	0.009	0.013	0.014
	N	15	15	15	15
Average of Cat 2	Pearson Correlation	0.647*	1	0.685**	0.409
	Sig. (2-tailed)	0.009	–	0.005	0130
	N	15	15	15	15
Average of Cat 3	Pearson Correlation	0.622*	0.685**	1	0.537*
	Sig. (2-tailed)	0.013	0.005	–	0.039
	N	15	15	15	15
Average of Cat 4	Pearson Correlation	0.620*	0.409	0.537*	1
	Sig. (2-tailed)	0.014	0.130	0.039	–
	N	15	15	15	15

** Correlation is significant at the 0.01 level (2-tailed)

* Correlation is significant at the 0.05 level (2-tailed)

### Design and evaluation of the mobile application

The findings from this step will be detailed in a future study. This mobile application offers general recommendations based on clinical guidelines.

## Discussion

To design a user-friendly self-care application with maximum efficiency, it is necessary to identify the content of this mobile-based application scientifically. For this purpose, a systematic review of CDSSs in the field of PCOS and designed mobile-based applications is needed [12–18]. While the first step of our research, previously published, focused on a systematic review of the literature and existing mobile applications related to PCOS [5], the current study advances the process by addressing steps two and three. These steps involve the development of a structured needs assessment tool and the validation of app requirements through expert input. The findings offer a practical and evidence-informed foundation that will directly support the design and evaluation of a user-centered mobile application in the next phase of the research. In the current study, we identified the requirements for designing a mobile application aimed at providing nutritional recommendations. These requirements were categorized into four groups: demographic information, data requirements, educational needs, and application capabilities. Out of the 75 identified items, 56 were determined to be essential for the application.

Key features of the application included recommending meals, food supplements, and foods based on their glycemic index (GI), all of which align with the functionality of existing applications. New features were also introduced, such as recommending food alternatives, suggesting meals based on disease symptoms, providing meal recommendations based on weight, suggesting drinks, advising on the elimination of harmful foods, and recommending healthy snacks. These enhancements could significantly improve the nutrition recommendation application.

In the study regarding educational needs, it was found that all items, except for “news of the Iran PCOS Society” and “experiences of patients with PCOS,” were considered necessary by the experts. The expert panel rated these two items as low-necessity, likely due to their limited direct impact on dietary behavior change or personal health management, which were the primary focus areas of our proposed application. In the study conducted by Sheikh Taheri et al. [9], an application with a self-care approach was developed based on two major requirements: educational needs and necessary skills. Among the 28 educational components, “causes of disease” were deemed unnecessary. However, in the present study, experts identified this component as necessary (CVR = 73%). Hajivandi et al. [19] reported that promoting healthy nutritional behaviors through an educational intervention program can improve the nutritional health of individuals with PCOS. Our study identified several essential educational needs related to PCOS, emphasizing the importance of prevention and healthy eating.

In the study conducted by Sinem Aslan et al. [20], deep learning algorithms were used to accurately estimate the caloric content of various foods. The researchers concluded that calorie counting should be excluded from the application, as they observed a decline in specialist referrals and an increase in false therapeutic beliefs related to calorie counting. Consequently, this feature was removed from the questionnaire. In the current study, 18 essential features for the mobile application were identified. Experts indicated that seven items related to drug consumption were considered less necessary.

In the study conducted by Sheikh Taheri et al. [9], similar to our current research, there was no section dedicated to providing drug recommendations. However, six essential items focused on managing drug consumption were included. Additionally, Rajdeep Kaur et al. [21] mployed Convolutional Neural Networks (CNN) to identify nutrients in food, suggesting that this approach could help patients with PCOS consume essential nutrients. In contrast, our study did not utilize any algorithms or AI techniques; instead, we focused on identifying the necessary features for designing and developing a nutrition recommendation system. We identified nine features related to food consumption that align with the objectives outlined by Rajdeep Kaur et al. [21], such as healthy food recommendations, advice for eliminating unhealthy foods, and dietary suggestions based on digestive health, symptoms, and body weight.

In another study, Rajdeep Kaur et al. [22] developed an artificial intelligence framework to help manage weight in patients with PCOS. In the research conducted by Sheikh Taheri et al. [9], it was noted that, in terms of nutrition and diet management, certain capabilities, such as “registration of daily food consumption” and “the ability to compare nutritional status with set goals,” need further investigation. However, the study did not provide specific nutritional recommendations tailored to the patient’s condition. In this study, we aimed to identify the essential requirements for designing a nutritional recommendation system that can guide patients in adopting a healthy eating pattern. Effectively managing eating habits can aid in weight management for these patients and also improve other symptoms and complications associated with PCOS.

In one study, Shanmugavadivel et al. [23] developed an AI-based diagnostic system for classifying patient and healthy referrals. This study used two types of clinical datasets and ultrasound image sets. According to the performance evaluation of machine learning models, the Support Vector Machines (SVM) model achieved the highest accuracy with 94.44%. The most important clinical features extracted were follicles, hair growth, weight gain, and ultrasound images for automatic diagnosis. This study proved that these diagnostic methods reduce the number of clinical tests and workload and save time. In the present study, our goal was to determine the requirements for the design and development of a recommender system focusing on patient nutrition, while the goal of the study above was to create a diagnostic system for PCOS patients. The use of SVM and machine learning models was also seen in other similar studies for prediction and classification. Zad et al. [24] proposed a machine learning approach (including SVM) for PCOS prediction using patient health records. Rajpurkar et al. [25] demonstrated the application of deep learning and SVM in diagnostic image classification for pneumonia detection. Esteva et al. [26] used machine learning models for skin cancer classification, showing comparable performance to dermatologists. The use of AI (such as SVM) in this study has not yet been implemented, but the studies [27] show that it has potential in PCOS-related applications.

Our study stands out due to its specific focus on “nutrition,” which distinguishes it from similar research, such as the studies conducted by Percy et al. [10]. In their work, nutrition is often considered just one aspect of a broader lifestyle. In contrast, our paper presents a more innovative approach by emphasizing nutrition as the primary focus for women with polycystic ovary syndrome (PCOS).

In terms of methodology, utilizing health experts through the Delphi method or a specialized survey with a needs assessment approach aligns with the strategy of Muhamad Jamil et al. [28], thereby reinforcing the scientific validity of the study. Similar to our study, this study used the CVR index and expert opinion. Our recommendations align with related studies [29, 30] regarding user interface simplicity.

Study limitations: This study has several limitations. Notably, it lacks real user (patient) involvement, and it employs a cross-sectional design. In similar studies focused on needs assessment in program development, researchers typically start by gathering input from health professionals before moving on to later stages [31]. Like previous mHealth design studies [32], we used a cross-sectional approach in the needs assessment phase. This approach is commonly applied in early-stage development to identify user and expert requirements before proceeding to the prototyping and validation phases. The academic levels of the respondents—faculty members versus PhD students—may contribute to differing viewpoints. Faculty members often rely on years of clinical or teaching experience, while PhD candidates typically offer fresher perspectives influenced by recent literature or exposure to digital health. While this diversity can enrich our findings, we acknowledge that it may also introduce biases stemming from different perspectives. For future studies, a longer duration should be considered, along with the inclusion of expert and patient feedback, to ensure that the minimum necessary data are collected more accurately.

## Conclusion

In this study, we identified 56 essential requirements in four categories for designing and developing a nutrition recommender mobile application for patients with PCOS, based on expert opinions. Given the rise in smartphone usage in recent decades, this study serves as a practical guide for nutritionists, researchers, and developers of health-focused mobile applications.

## Data Availability

All data generated or analyzed during this study are included in this manuscript.

## Abbreviations

PCOS: Polycystic ovary syndrome
GI: Glycemic index
CDSS: Clinical decision support system
BMI: Body mass index
CVR: Content validity ratio
CNN: Convolutional neural network
SVM: Support vector machines
